# Robust discrimination of multiple naturalistic same-hand movements from MEG signals with convolutional neural networks

**DOI:** 10.1162/imag_a_00178

**Published:** 2024-05-20

**Authors:** Ivan Zubarev, Mila Nurminen, Lauri Parkkonen

**Affiliations:** Department of Neuroscience and Biomedical Engineering, Aalto University School ofScience, Espoo, Finland; AaltoNeuroImaging, Aalto University, Espoo, Finland

**Keywords:** convolutional neural network, magnetoencephalography, brain–computer interface, motor imagery, motor rehabilitation, interpretability

## Abstract

Discriminating patterns of brain activity corresponding to multiple hand movements are a challenging problem at the limit of the spatial resolution of magnetoencephalography (MEG). Here, we use the combination of MEG, a novel experimental paradigm, and a recently developed convolutional-neural-network-based classifier to demonstrate that four goal-directed real and imaginary movements—all performed by the same hand—can be detected from the MEG signal with high accuracy:>70%for real movements and>60%for imaginary movements. Additional experiments were used to control for possible confounds and to establish the empirical chance level. Investigation of the patterns informing the classification indicated the primary contribution of signals in the alpha (8–12 Hz) and beta (13–30 Hz) frequency range in the contralateral motor areas for the real movements, and more posterior parieto–occipital sources for the imagined movements. The obtained high accuracy can be exploited in practical applications, for example, in brain–computer interface-based motor rehabilitation.

## 1 Introduction

Motor imagery with brain–computer interface (BCI)-based feedback has emerged as a promising approach in motor rehabilitation following, for example, cerebral stroke. Such restorative BCIs rely on the ability of patients to modulate their brain activity intentionally using, for example, attempted movements or motor imagery. If such modulations can be reliably detected by non-invasive measurements, one can envision a system where an effector device linked to a detector assists patients in producing these otherwise impaired movements. Repeated use of such a system can promote neurological recovery by inducing neuroplasticity in the affected brain circuits (Cervera et al., 2018;Girges et al., 2022;Nojima et al., 2022;Soekadar et al., 2015).

Neural mechanisms underlying the BCI-induced improvement of motor function are not fully understood but are generally hypothesized to be based on the principles of operant conditioning (Soekadar et al., 2015). Importantly, operant conditioning literature suggests that the optimal reinforcement involves a degree of uncertainty to establish a lasting association between the behavior and the reinforcer. Such a varying rate of reinforcement is known to make the learned behaviors resistant to extinction (Herrnstein, 1961). In the BCI domain, the optimal reinforcement has been implicitly determined to take place when the feedback is delivered at 70–80% accuracy. Existing non-invasive restorative BCI systems achieve this accuracy only by utilizing a single movement per limb, for example,Irimia et al. (2016). Here, we investigate if such a high level of discrimination accuracy can be achieved when training multiple movements, performed with the same hand.

To this end, we combine (1) a novel experimental paradigm based on naturalistic movements, (2) state-of-the-art magnetoencephalography (MEG), and (3) an end-to-end convolutional neural network-based decoder capable of combining multiple signal features.

It has been suggested that spatial organization of the motor cortex may follow the ethologically-valid synergistic movements (Brown & Teskey, 2014;d’Avella et al., 2003;Graziano, 2016;Leo et al., 2016;Pereira et al., 2017). Here, we hypothesize that using such complex, goal-directed movements in the BCI context can be beneficial in several ways. First, realistic, goal-directed movements could be easier to imagine (especially when providing the context) compared with isolated movements (e.g., ulnar/radial rotation, extension/flexion) used previously in similar tasks. Second, we hypothesize that the representation areas of these movements may overlap less in the sensory-motor cortex, leading to better discrimination of the corresponding spatiotemporal patterns from the MEG signal.

Multiple distinct neurophysiological signatures are present during movement intention, preparation, imagery, and execution. Apart from the movement-related evoked responses, modulations of oscillatory activity in the alpha (8–12 Hz) and beta (13–30 Hz) frequency range occurring roughly 200 ms prior to the onset of movement have been linked to the kinematic outcome of the planned movement (Engel & Fries, 2010;Nakayashiki et al., 2014;Schnitzler et al., 2006). Similarly, activity in the gamma frequency range (30–60 Hz) occurring up to 700 ms after the movement onset (Cheyne et al., 2008;Nowak et al., 2018;Szurhaj et al., 2006) linked mainly to processing the proprioceptive afferentation from the muscles and joints involved in the movement (Kolasinski et al., 2020). Moreover, it has been established that observing movements induces changes in the activity of the sensorimotor system comparable with those occurring during action execution (Hari et al., 1998;Meirovitch et al., 2015).

Despite their higher costs, MEG-based BCIs can be used in a hospital setting, provided that the higher spatial resolution of MEG can provide additional benefits for the rehabilitation process (Buch et al., 2008;Mellinger et al., 2007). One such benefit could be the ability to resolve multiple intended or imagined movements from the same limb, leading to more versatile and engaging training protocols and, thus, better clinical outcomes. Here, we demonstrate that up to four different real or imaginary movements of the same hand can be decoded with high accuracy, sufficient for practical applications, for example, motor rehabilitation. Thus, the key contribution of this study is combining (1) an original experimental paradigm focused on naturalistic movements with (2) a novel convolutional-neural-network-based classifier developed specifically for MEG signals (LFCNN) (Zubarev et al., 2019). One advantage of the CNNs is that they do not rely on manual feature extraction, and thus learn informative features from the data directly. Because LFCNN architecture closely follows the generative model of the MEG signal, it also allows exploration of the patterns, it learns from the data and uses it to discriminate between different movements. Finally, (3) we use discriminative pattern interpretation analysis, standard MEG analysis, and two control experiments to confirm that the classification between different movements was driven primarily by the brain activity originating from the contralateral somatomotor cortices within the frequency range of theα(8–12 Hz) andβ(13–30 Hz) rhythms.

## 2 Materials and Methods

### 2.1 Participants

In total, 12 healthy right-handed volunteers (7 male, 5 female, mean age30.4±5.1years) performed 4 different real and imaginary movements with their right hand, following visual cues displayed on the screen. The data from one subject were discarded from the analysis due to the fact that the participant was repeatedly falling asleep during the measurement session. Additionally, the data from one subject were discarded due to excessive levels of noise. All participants were familiarized with the experimental procedure by reading the printed instructions and signing the informed consent form. The study was approved by Aalto University Committee on Research Ethics.

### 2.2 Experimental procedure

At the beginning of each trial, a visual cue indicating the situation where the target movement is performed was shown on the screen. Additionally, a 1.5-second time bar was shown below the cue. The time bar was shrinking linearly and participants were instructed to initiate the movement or imagery when the bar disappeared completely. At that moment the visual cue was replaced by the image of the target movement, this was done to prevent the participants from shifting their gaze to their hand during the movement blocks and, additionally, to facilitate the imagery during the imagery blocks. At the end of each trial, participants were instructed to return their hands to the starting neutral position. The experimental procedure and the visual cues used as stimuli are shown inFigure 1.

**Fig. 1.**
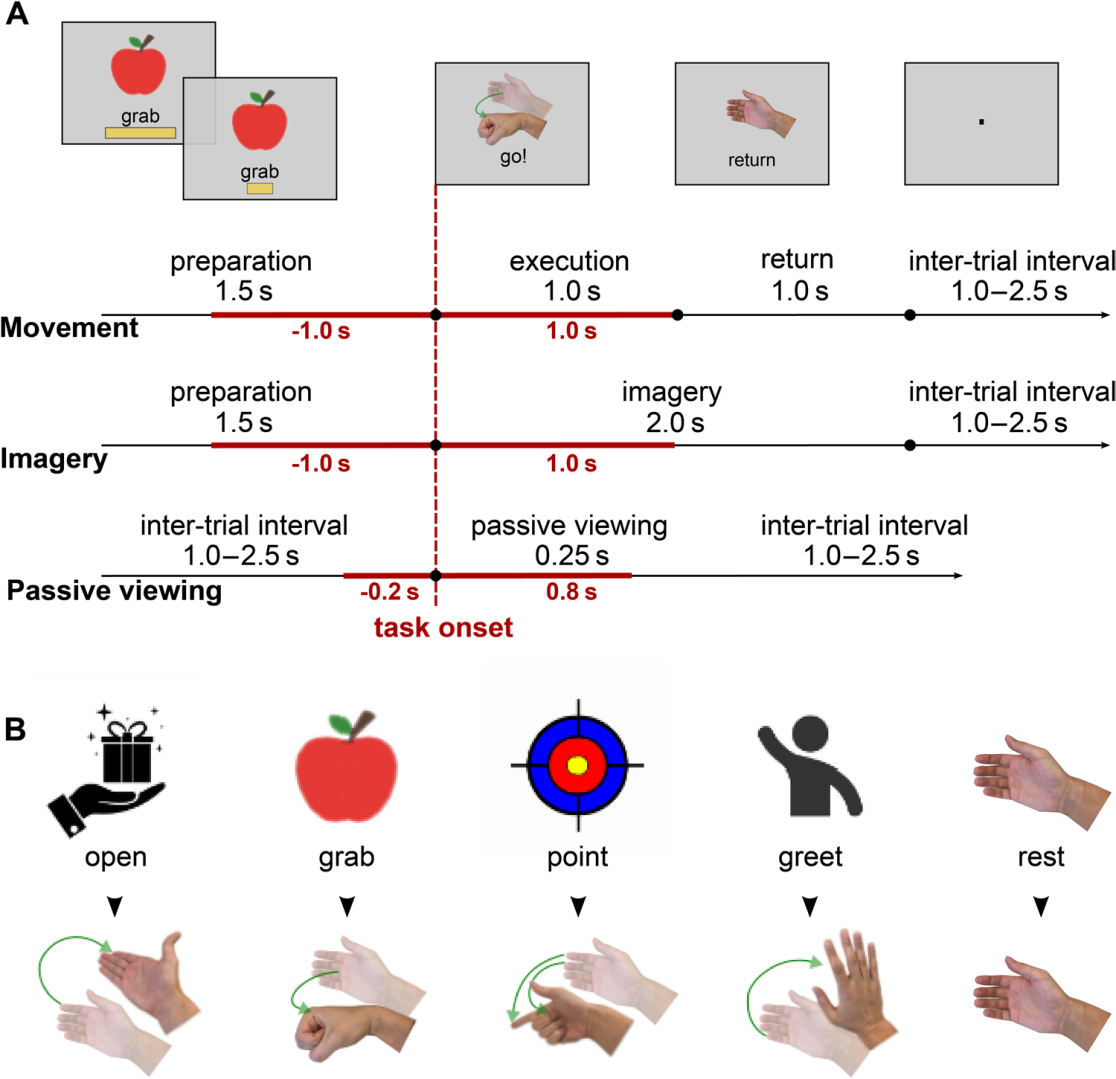
Experimental procedure and timing. (A) Each trial started with a visual cue and a shrinking time bar appearing on the screen. Subjects performed movement or imagery when the time bar had disappeared completely. After performing real movements, subjects were instructed to return the hand to the starting position after 1 second. Red lines indicate the time windows used for the decoding analysis. (B) Visual cues for movement preparation (left) and the corresponding movements (right).

The subjects were sitting comfortably in the inclined position (60 degrees) with their right hand positioned on the cushion in front of them approximately against the midline of the body with the thumb facing upward at about 45 degrees, allowing for roughly the same range of motion for movements involving ulnar and radial rotation.

The experiment consisted of 6 blocks (3 real-movement blocks and 3 imagined-movement blocks) with each block comprising 20 repetitions of the task. Each block lasted for 12 minutes with short breaks in between. These experimental blocks were presented in an alternating order with a real-movement block always presented first. This procedure resulted in a total of 270 trials per the real and imagined movement condition, comprising 60 repetitions of each of the 4 movements and, additionally, 30 “rest” trials. During the analysis, these “rest” trials were combined across the real and imagined movement conditions, resulting in a total of 60 repetitions and thus a balanced trial count per each category, and were used as an additional class in both decoding experiments.

In the control experiment, the participants were presented with the same cue images but were instructed to neither perform nor imagine the displayed movement. This control was done to establish the empirical chance level and to verify that the classification results were not driven by the evoked responses to the visual stimuli alone; seeFigure 1.

### 2.3 Measurements and preprocessing

MEG data were filtered to 0.01–330 Hz and collected continuously at a 1000-Hz sampling rate using the 306-channel MEGIN Triux Neo MEG system (MEGIN Oy, Espoo, Finland) comprising 102 magnetometers and 204 planar gradiometers. Continuous head position tracking was performed using five HPI coils placed on the forehead and behind both ears. A single bipolar electrooculographic electrode pair was placed on the outside edge and below the right eye to monitor vertical and horizontal eye movements. Additionally, two accelerometers were placed on the index and ring fingers of the right hand to track the hand kinematics during the movement blocks and to ensure the lack of residual movements during the imagery blocks.

The data were preprocessed using the MaxFilter software (version 2.2; MEGIN Oy, Espoo, Finland) to suppress external magnetic interference and to compensate for head movements using the temporally-extended signal-space separation (tSSS) method (Taulu & Simola, 2006). Following tSSS, the MEG data were band-pass filtered to 0.1–90 Hz and downsampled to 500 Hz. Cardiac and oculomotor artifacts were suppressed by removing up to two independent components identified by visual inspection (Gramfort et al., 2013;Hyvärinen & Oja, 2000). Only the data measured by the planar gradiometers were retained for further analysis. MEG epochs comprising 1 second before and 1 second after the expected movement (or imagery) onset were extracted and scaled by subtracting the mean and dividing by the standard deviation of the whole 2-second epoch across all channels for each epoch independently. Movement onset was determined separately for each trial based on the root-mean-squared magnitude of hand acceleration. Whenever the combined acceleration exceeded the standard deviation of the pre-stimulus period by a factor of 10, was considered as the movement onset (21.3 ± 176.1 ms mean latency with respect to the onset of the visual cue signaling to initiate the movement). No such realignment was performed for motor imagery.

### 2.4 Decoding experiments

#### 2.4.1 Hyperparameter search

We performed a limited hyperparameter search for the LFCNN classifier. The hyperparameters were chosen, so that they maximized the cross-validation performance on the data from the movement experiment and then applied to the imagery, across-subject, and control experiments as is. The explored hyperparameter space and the optimal values are summarized inTable 1.

**Table 1.** Tested and optimal hyperparameter values for LFCNN classifier.

Parameter	Tested	Final
Number of latent components	32	32
Kernel size	32,64,128,256	32
Pooling factor	16,32,64,128	64
Pooling stride	8,16,32,64	32
Pooling type	max, avg	avg
Non-linear activation function	ReLU	ReLU
Drop-out rate	0.25,0.5	0.5
$l_{1}$ -penalty	$3\cdot 10^{-3}$ , $3\cdot 10^{-4}$ , $3\cdot 10^{-5}$	$3\cdot 10^{-3}$
Minibatch size	5,25,50	50
Training iterations per epoch	25,50,100	25
Learning rate	$1\cdot 10^{-4}$ , $3\cdot 10^{-4}$	$3\cdot 10^{-4}$

#### 2.4.2 Within-subject decoding

**Fig. 2.**
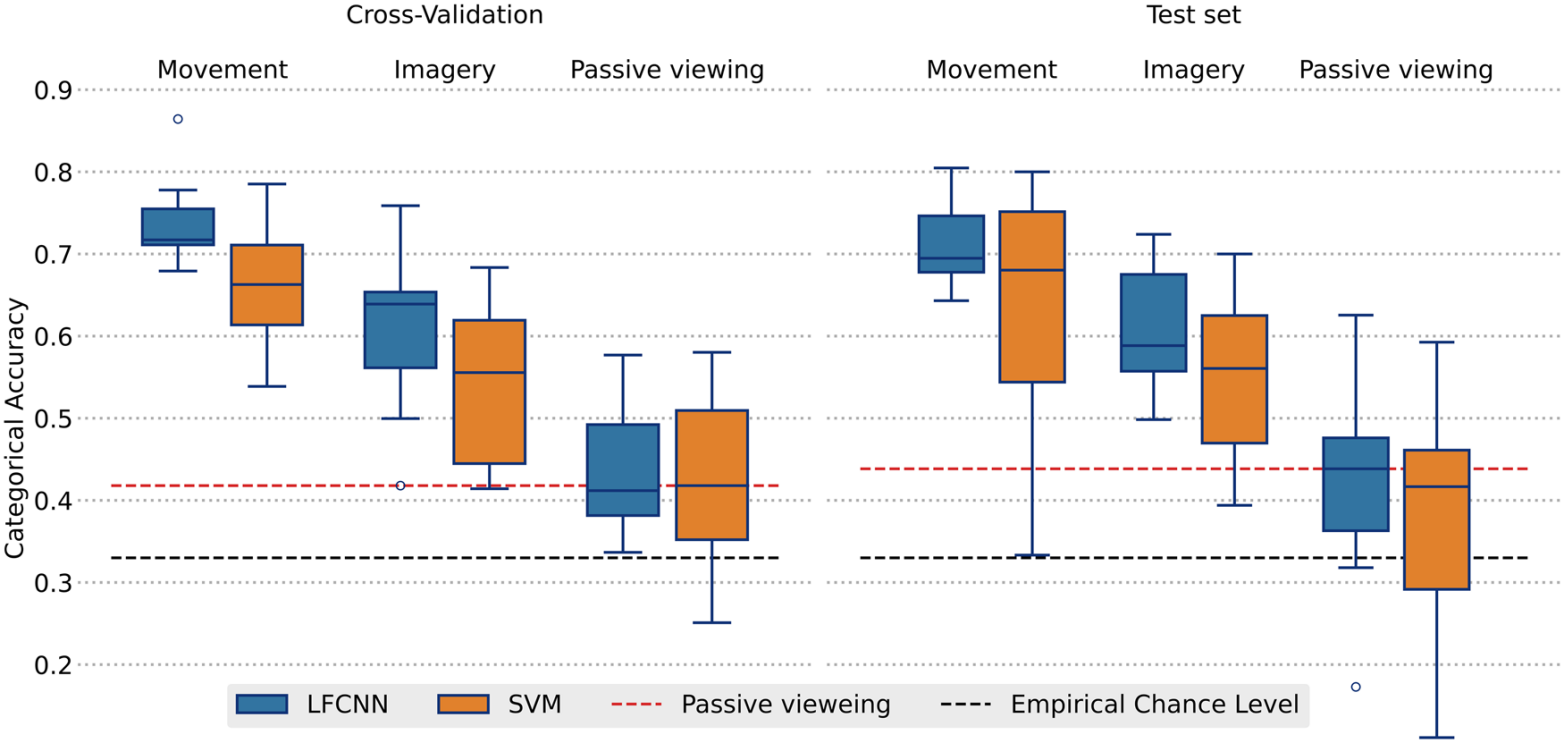
Within-subject (nine-fold nested cross-validation) classification results. The box height indicates the interquartile range. Whiskers indicate the full data range, excluding a single outlying data point. Horizontal lines inside the box indicate the median. Empirical chance level (black dashed line) for a four-class problem with n = 300. The de-facto chance level (red dashed line) was defined as the mean cross-validation accuracy obtained in the control passive viewing experiment.

The preprocessed MEG 2-second epochs centered on the movement (or imagery) onset were used to train an interpretable Convolutional Neural Network-based model introduced inZubarev et al. (2019)as implemented in MNEFlow software (Zubarev et al., 2022) using Tensorflow (Abadi et al., 2016). The data of each participant were split randomly into 10 folds. Model performance was estimated using a nested nine-fold cross-validation scheme where on each fold the model was trained on 80% of the data, 10% of the data were used as a validation set used for tracking model convergence and early stopping. Out-of-sample performance was estimated on the remaining 10% of the data (holdout test set). We report average categorical accuracy across nine folds following a standard cross-validation approach (cross-validation accuracy) along with the accuracy on the holdout set, averaged across the nine training folds (test accuracy). Each training epoch comprised 25 training steps performed on mini-batches of 50 MEG epochs. For each fold, the model was trained using Adam optimizer with a learning rate of$3\cdot 10^{-4}$until the early stopping criteria were met, that is, the validation loss did not decrease for 10 consecutive training epochs. After the training was complete, the model was restored to the state yielding minimal validation loss. The confusion matrix and the five-class categorical accuracy for that model were estimated on the validation fold, and stored as intermediate performance estimates for each fold. The final results (Fig. 2) represent the average categorical accuracy across all nine folds estimated on the validation and test sets for each participant separately.

**Fig. 3.**
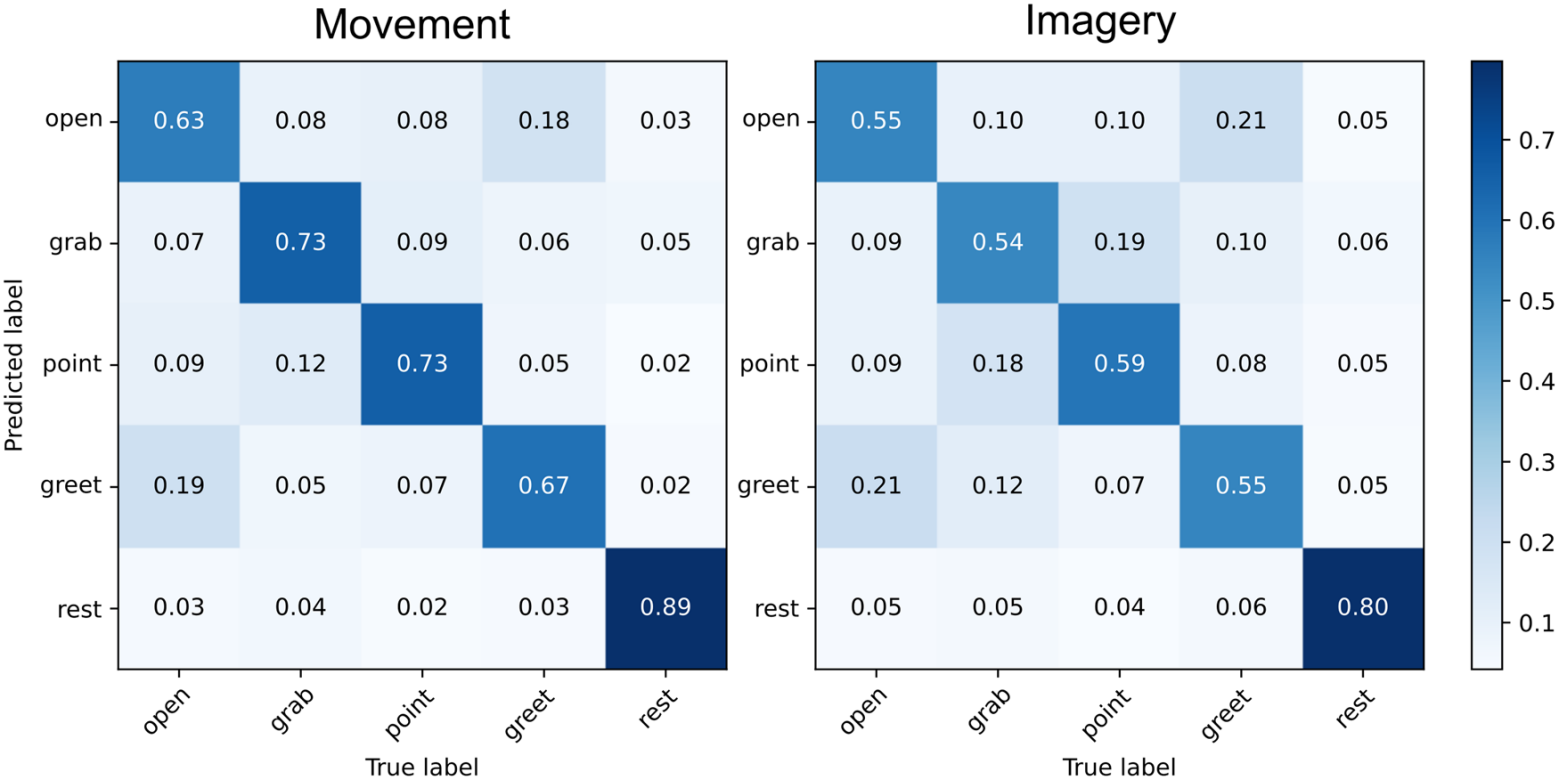
Normalized confusion matrices in movement and imagery experiments combined from all subjects and all validation folds.

Grand-average confusion matrices (Fig. 3) were obtained by summing the confusion counts matrices estimated on each validation fold for each participant, and normalizing by the total trial count in each class.

Additionally, we estimated the performance of the Support Vector Machine (SVM) classifier with radial basis function kernel as a baseline model. We performed a grid hyperparameter search for regularization coefficient (C) ranging from$1\cdot 10^{-4}$to$1\cdot 10^{4}$for each subject individually. We report the highest cross-validation accuracy achieved in nine-fold cross-validation as well as out-of-sample prediction accuracy on the held-out test set comprising 10% of the data.

#### 2.4.3 Across-subject decoding

**Fig. 4.**
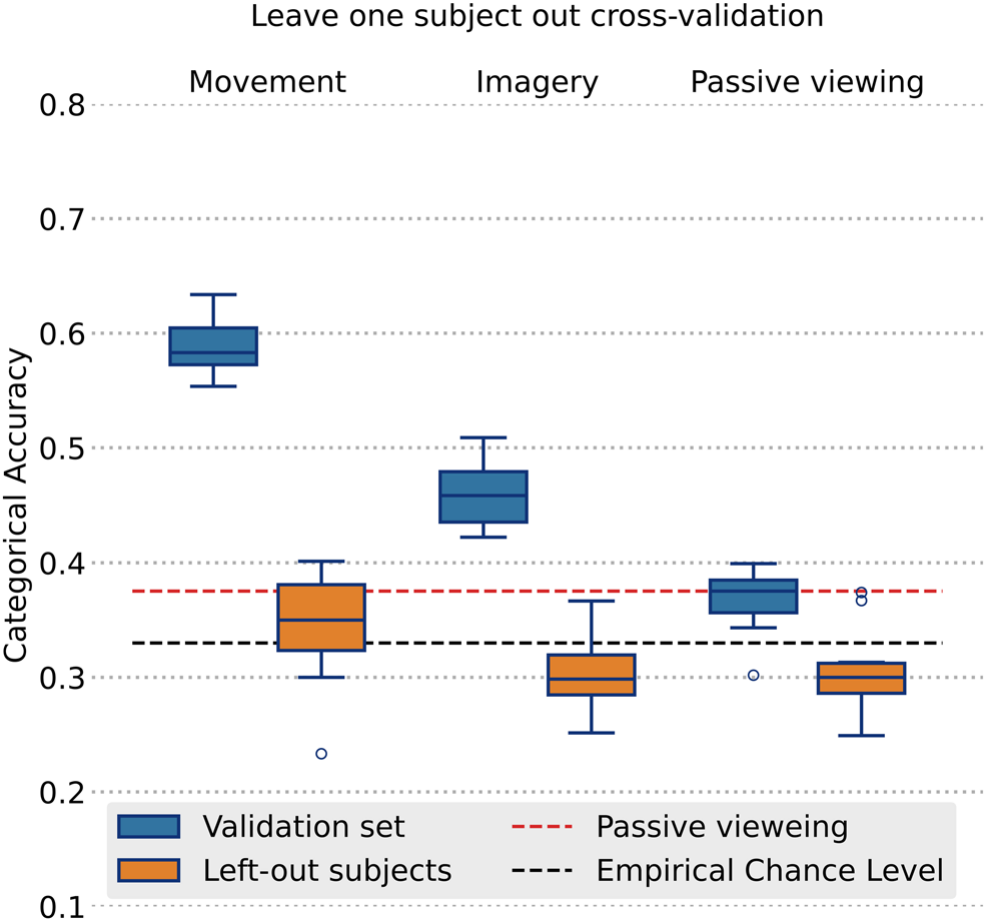
Across-subject (leave-one-out cross-validation) classification results. The box height indicates the interquartile range. Whiskers indicate 150% of the interquartile range. Horizontal lines inside the box indicate the median. Empirical chance level (black dashed line) for a four-class problem with n = 300. The de-facto chance level (red dashed line) was defined as the highest mean cross-validation accuracy obtained in the control experiment.

To test if patterns of activity corresponding to each movement were similar across subjects, we performed an across-subject decoding experiment using the leave-one-subject-out (LOSO) cross-validation approach. On each fold, the model was trained on pooled data of all subjects except one. After the training was complete, the model performance was tested by computing categorical accuracy when predicting the movement (or imagery) type on the held-out subject. This procedure was repeated 10 times so that each subject was used as a held-out subject. The final results represent average predictive performance on thus held-out subjects. In this LOSO experiment, 10% of the pooled training data were used as a validation set to track model convergence. Hyperparameter values and training procedures were identical to the ones used in within-subject decoding. Similarly to the within-subject experiment, we report average categorical accuracy on the validation set (comprising the data from participants included in the training set), as well as the average accuracy obtained on the holdout subjects (Fig. 4).

### 2.5 Pattern interpretation

To investigate patterns of the MEG signal that allowed the discrimination between different movements, we explored the model weights to obtain spatial and spectral properties of the components that informed the classification.

Contributions ofk-th latent component toj-th class were defined as an average activation of all nodes of the final layer corresponding to this component.

$$c_{kj}={\dot{{\text{w}}_{kj}^{out}}}^{T}{\text{h}}_{kj}^{tconv}+b_{j}^{out}.$$ (1)

Spatial activation patterns of each latent component were obtained by multiplying each corresponding spatial filter by the spatial covariance matrix of the data (Haufe et al., 2014).

$${\text{a}}_{k}=\Sigma^{T}\dot{{\text{w}}_{k}^{dmx}}$$ (2)

Spectral properties ofk-th latent component were obtained by the following procedure. First, we computed the average power spectral density of the time course obtained by applyingk-th spatial filter onto each sample of the validation set. The resulting power-spectral density estimate was then multiplied by the frequency response of thek-th temporal convolution kernel, and divided by its total power to obtain the estimate of the spectral fingerprint of the corresponding time-course following the procedure suggested inPetrosyan et al. (2021).

Finally, the combined pattern corresponding to the conditional mean of each class was obtained by computing a sum of individual spatial and spectral patterns of each latent component weighted by the contribution of this component to this class (Kindermans et al., 2018). This procedure resulted in a single combined pattern for each class and was performed for each fold of the five-fold cross-validation. The final patterns for each class were obtained by averaging the obtained patterns across the folds.

#### 2.5.1 Source estimation

We selected two subjects and performed a source estimation for the spatial activation patterns obtained for each class using the procedure described above. Individual T-1 weighted structural MR images were obtained for two subjects and used to build the forward model using the boundary-element method. The noise covariance matrix was estimated using the data from the inter-stimulus interval (from 2.5 seconds to 1.5 seconds prior to the movement onset) using Ledoit-Wolf shrinkage approach as implemented in MNE-Python software (version 1.2.1) (Gramfort et al., 2013). Inverse solutions were obtained using Minimum-Norm Estimation method (Hämäläinen & Ilmoniemi, 1994) with dipole orientations loosely constrained to normal orientation to the cortical surface (0.3) with no depthweighting.

### 2.6 Standard MEG analysis

#### 2.6.1 Preprocessing

MEG data were preprocessed as described above and segmented into epochs from — 2.5 to 3.0 seconds with respect to task onset (movement onset in real movement trials and onset cue in imaginary movement and rest trials) using MNE-Python software (version 1.2.1) (Gramfort et al., 2013). The epochs were baseline corrected by subtracting the mean signal of the baseline period (from 2.3 to 1.8 seconds prior to the movement (or imagery) onset) from the entire epoch.

#### 2.6.2 Evoked response analysis

**Fig. 5.**
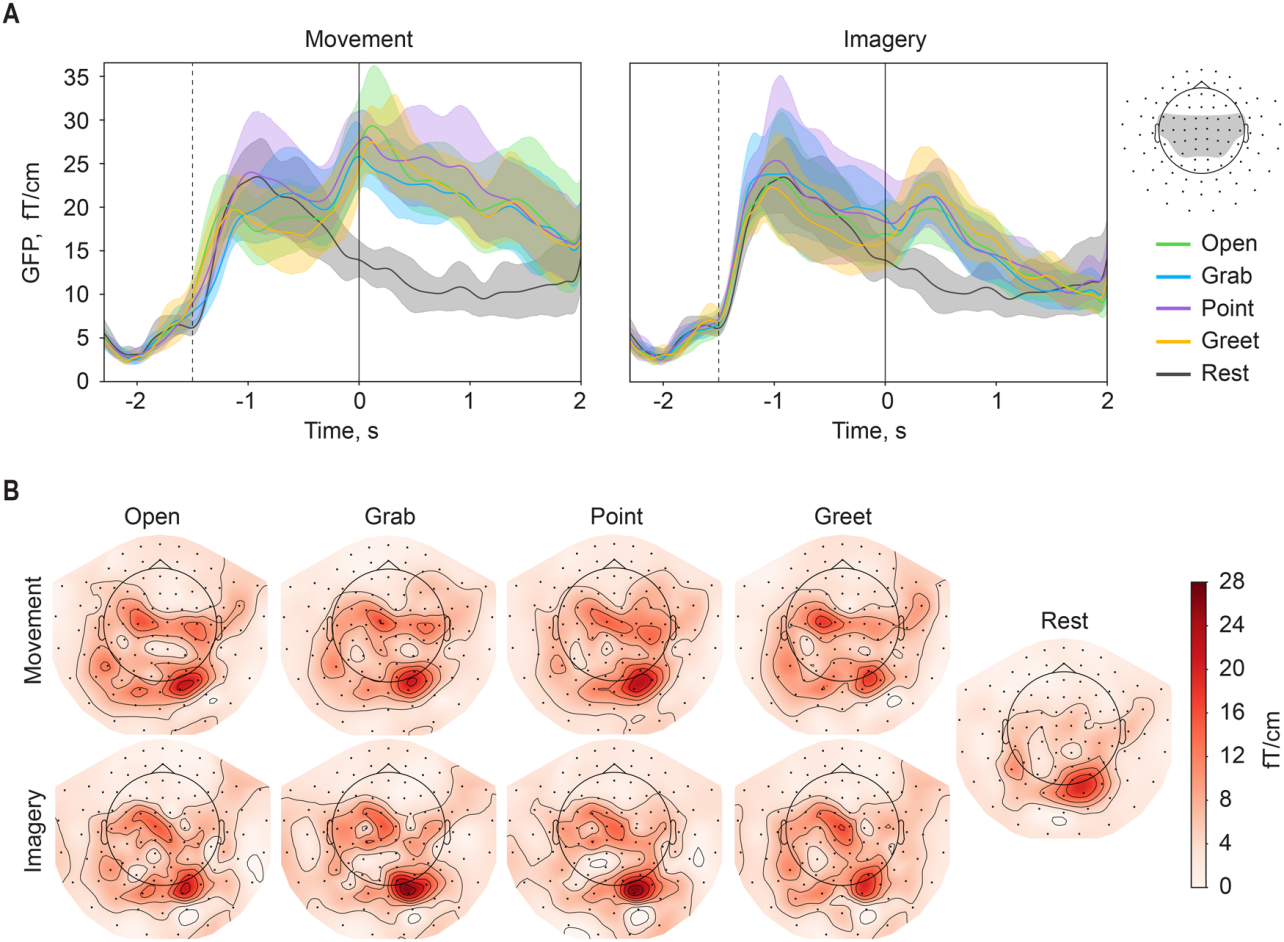
(A) Global field powers of the real and imagined movements across 52 channels over the vertex area (shaded channels in top right). Transparent lines denote 95% confidence interval for the grand average. Vertical lines indicate the onset of the visual cue (dashed) and the task onset (solid). For real movements, the timing of the visual cue is the average timing relative to the movement onset determined by the accelerometers. (B) Topographical distribution of task-related evoked fields. Activity is averaged from 1 second before to 1 second after the task onset. All values are grand-averaged over nine participants.

Global field power (GFP) over a cluster of 52 central and parietal gradiometers was calculated across all participants. The topographical distribution of the phase-locked activity was estimated by averaging the GFPs over the whole 2 seconds time window centered on the task onset for each movement separately (Fig. 5). To highlight the contribution of low-frequency activity, the data were low-pass filtered with 3 Hz cutoff frequency. One subject was left out from the grand-average evoked field analysis, as the responses differed significantly from the other subjects, being strongly represented in the ipsilateral hemisphere (seeSupplementary Fig. S1).

#### 2.6.3 Time–frequency analysis

**Fig. 6.**
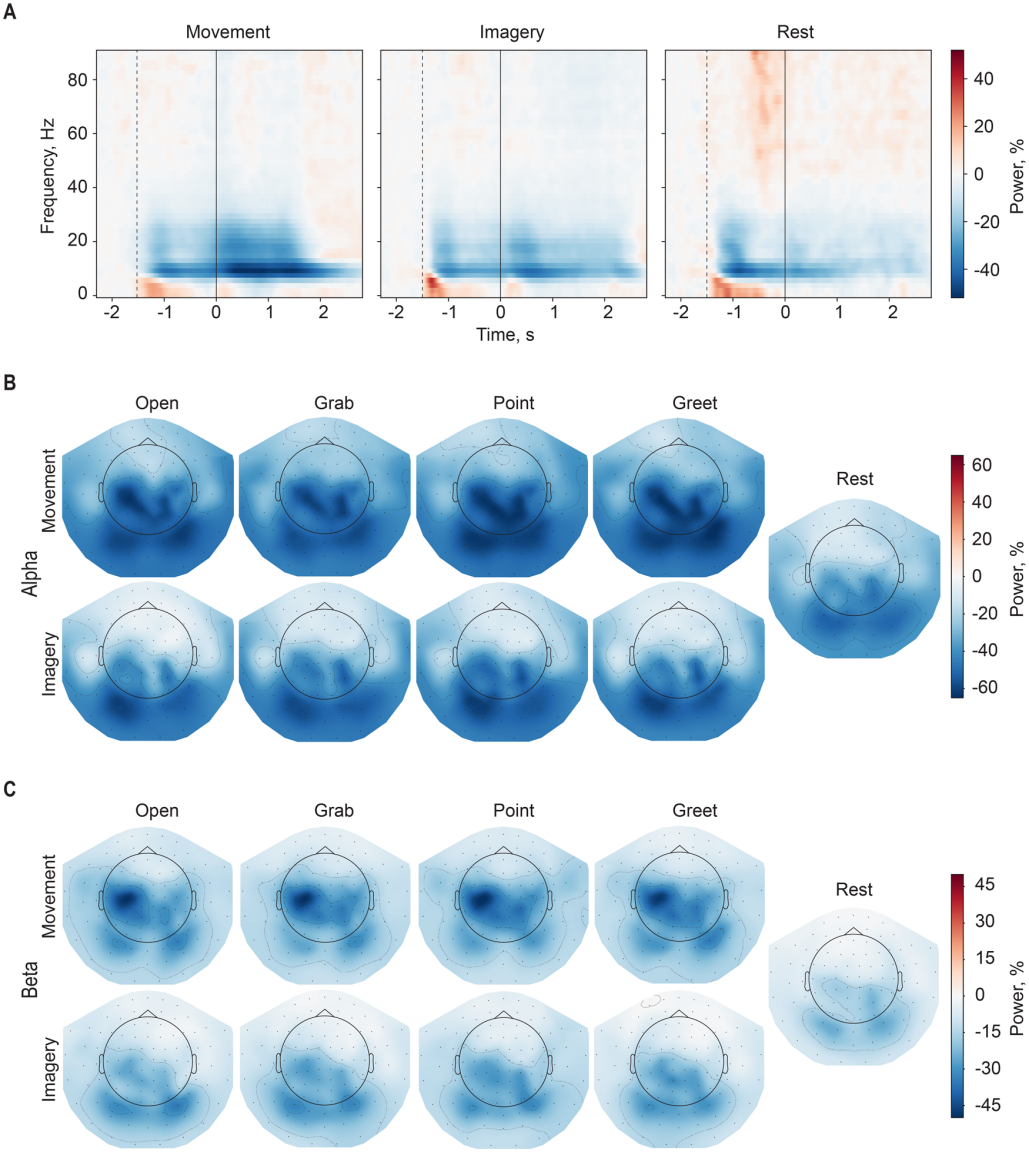
(A) Time–frequency representations of the real movements, imagined movements, and rest, data combined from all sensors. Vertical lines indicate the onset of the visual cue (dashed) and the task onset (solid). For real movements, the timing of the visual cue is the average timing relative to the movement onset determined by the accelerometers. (B) Topographical distribution of task-related alpha and (C) beta activity during the four real and imagined movements and rest. Activity is averaged from 1 second before to 1 second after the task onset. All values are grand-averaged over subjects and expressed as a percentage change from the baseline level (from 2.3 to 1.8 seconds before the cue).

The time–frequency responses (TFRs) for the 0.1–90 Hz frequency range of the whole epoch were computed using Morlet Wavelet with a frequency resolution of 1.8 Hz and a number of wavelet cycles proportional to wavelet frequency divided by 2. The first and last 0.2 seconds were cropped to remove the edge effects. Responses were first averaged across trials for each subject and movement type (executed movement, imaginary movement, and rest) separately and then averaged across subjects. Grand-averaged time–frequency responses of each movement type are presented inFigure 6as percentage changes from the baseline period. Based on observations in time–frequency analysis, grand-averaged topographical distribution of alpha (8–12 Hz) and beta (13–30 Hz) frequency band powers was produced separately for each movement in the 2-second time window centered on the task onset (Fig. 6).

## 3 Results

Ten healthy right-handed volunteers performed four different real and imaginary movements following visual cues as illustrated inFigure 1. Two-second epochs of MEG data centered on the task onset were used to train the LFCNN classifier.

### 3.1 Within-subject decoding

Model performance was estimated for each subject individually using a nine-fold nested cross-validation. The obtained grand-averaged cross-validated categorical accuracy using LFCNN was73.2±60.3%when decoding executed movements and60.0±9.5%when classifying imaginary movements. The mean out-of-sample performance evaluated on the held-out test set was71.1±6.9%for executed movements and61.3±8.4%for imaginary movements (Fig. 2). Detailed information is presented inTable 2. The baseline model (RBF-SVM) yielded mean cross-validated accuracy of66.4±7.0%for executed movements and54.3±9.8%for imaginary movements. Mean out-of-sample predictive performance was63.9±14.2%and54.5±9.8%, for executed and imaginary movements, respectively. Detailed information is presented inSupplementary Table S1. Both models performed significantly above the empirical chance level (paired Student’s*t*-test,p<0.001) defined in a separate control experiment.

**Table 2.** Categorical accuracy in within-subject decoding.

	Movement		Imagery		Accelerometers		Visual
Subject	CV	TEST	CV	TEST	Real	Imagined	Control
s01	72.0%	74.3%	66.5%	70.0%	99.0%	37.0%	27.0%
s02	70.4%	63.7%	52.3%	55.2%	97.7%	31.3%	22.0%
s03	80.3%	71.1%	68.1%	62.9%	99.7%	29.3%	34.3%
s04	73.2%	63.7%	54.2%	54.8%	99.7%	32.0%	47.3%
s05	64.7%	63.6%	59.1%	65.3%	99.7%	29.1%	29.7%
s06	66.7%	65.3%	40.3%	53.2%	98.5%	45.8%	35.2%
s07	87.9%	80.8%	77.0%	76.4%	99.4%	37.0%	54.8%
s08	74.6%	75.8%	63.4%	54.9%	97.0%	50.0%	27.3%
s09	70.8%	69.0%	56.5%	50.5%	98.8%	25.8%	31.5%
s10	71.5%	83.5%	63.2%	70.4%	100.0%	33.0%	21.2%
Mean	73.2%	71.1%	60.4%	61.4%	98.9%	35.0%	33.0%
s.d.	6.3%	6.9%	9.5%	8.4%	0.9%	6.9%	9.7%

The CV accuracies are from nine-fold cross-validation.

Inspecting the confusion matrices (Fig. 3) revealed that discriminating between different movement types was more challenging compared with separating the movement classes from the “rest” class. Therefore, we repeated the analysis using only four movement classes. The model trained in this four-class scenario resulted in predictive performance comparable with that observed in the five-class setting for both the movement (cross-validation accuracy –70.2±6.6%, test set accuracy –70.2±5.3%) and imagery tasks (cross-validation accuracy –61.6±9.2%, test set accuracy –58.6±8.1%). The detailed results of this four-class analysis are reported inSupplementary Table S3andSupplementary Figures S1 and S2. A series of paired*t*-tests confirmed that in the four-class scenario, the predictive performance was significantly higher in the movement and imagery conditions compared with the passive viewing condition (p<0.001).

### 3.2 Across-subject decoding

In a leave-one-subject-out experiment, the model was able to perform above chance on the pooled data from the subjects in the training set (mean validation accuracy for executed movements58.6±2.3%and46.1±2.8%for imaginary movements) but performed within the chance level when tested on the held-out subjects (mean accuracy for executed movements34.3±4.6%and30.5±3%for imagined movements), indicating that the observed movement-related patterns varied considerably across individuals (Fig. 4).

In the visual control experiment, subjects viewed the images depicting the target movement without imagining or performing these movements. In this condition, the model performed above the theoretical chance level of 20%, as well as the empirical chance level for a 4-class problem with n = 300 (Combrisson & Jerbi, 2015), but significantly below the accuracy level obtained for real and imagined movements, indicating that their classification cannot be based on the responses to the mere visual information instructing the movements.

In the second control experiment, the LFCNN classifier was applied to the accelerometer signals measured during the imagery blocks, where subjects were instructed not to perform any movements.

The results from these control experiments are summarized inTable 2.

#### 3.2.1 Pattern interpretation and standard analysis

In line with previous findings, time–frequency analysis indicated significant event-related suppression in theα- andβ-frequency ranges for all four movements. As expected, these effects were less pronounced for the imagined compared with the real movements. Interestingly, the spatial distribution of these effects was characterized by a more posterior focus for the imagined compared with the real movements. Overall, while all experimental conditions induced significant changes in the sensory-motor rhythms, visual inspection of their topographical distribution did not reveal notable differences between the movement types (Fig. 6).

**Fig. 7.**
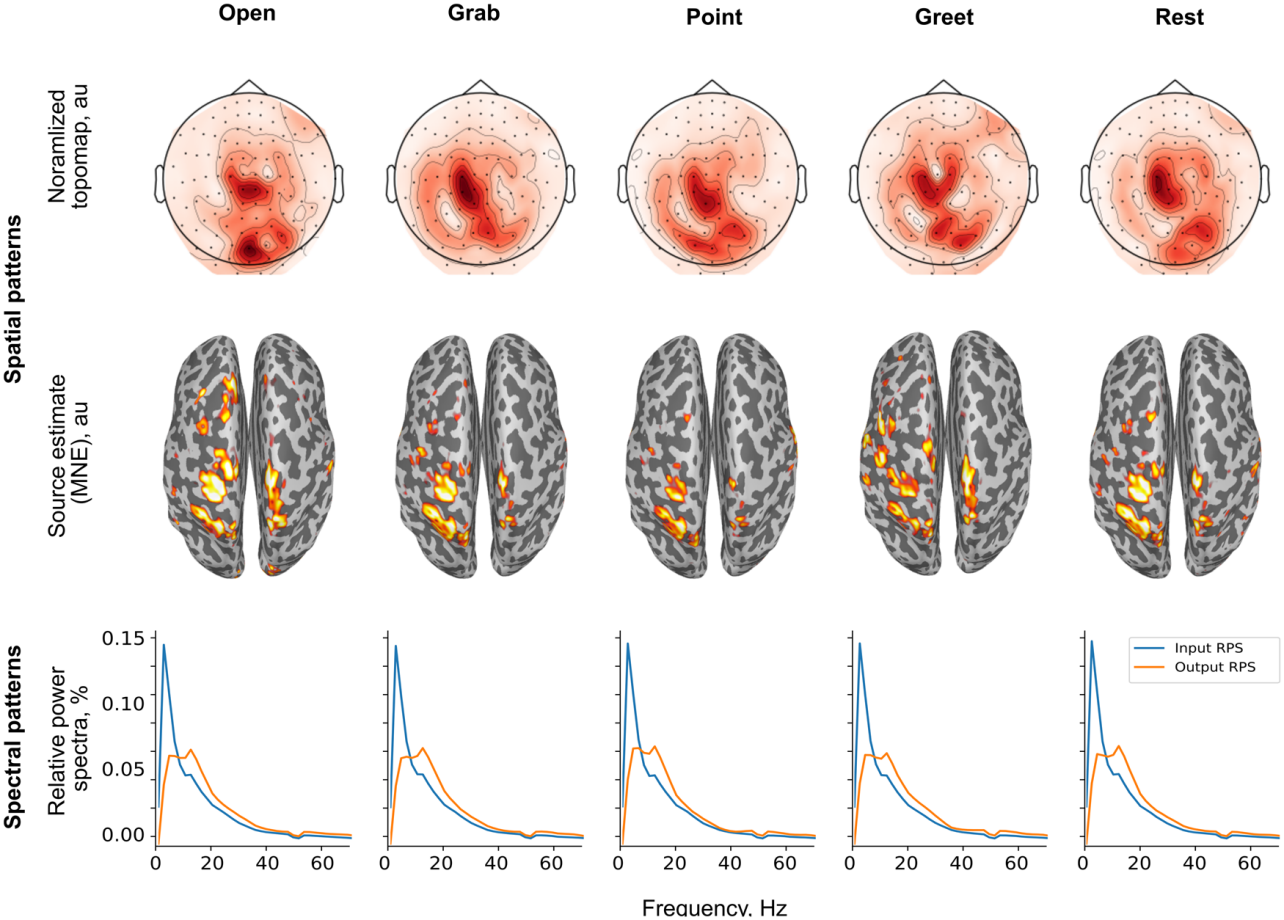
Spatial patterns discriminating the four real movements in a single subject (s08).**Top row:**Spatial activation patterns corresponding to the class-conditional mean of each condition in the sensor space (norm of planar gradiometer pairs).**Middle row:**Minimum-Norm source estimates of the spatial activation patterns with the top 10% of the estimate shown.**Bottom row:**Relative power spectra of the MEG signal after the spatial demixing layer (blue), and the estimated spectral fingerprint of the corresponding latent source (orange), both given as a percentage of the total.

Because across-subject decoding remained at the chance level, we limited the pattern interpretation to individual subjects.Figures 7and8show the discriminative patterns extracted from the trained CNN classifier for the real and imagined movements in representative subjects. Overall, in 7 out of the 10 subjects, these patterns for the real movements were focused over the contralateral central and parietal sensors, while the patterns extracted for the imagined movements were characterized by posterior foci over occipital areas (in 8 out of 10 subjects). The spectra of the informative components showed prominent peaks within theα−orβ−frequency range (real movements: in 8 out of 10 subjects, imagined movements: in 10 out of 10 subjects).

**Fig. 8.**
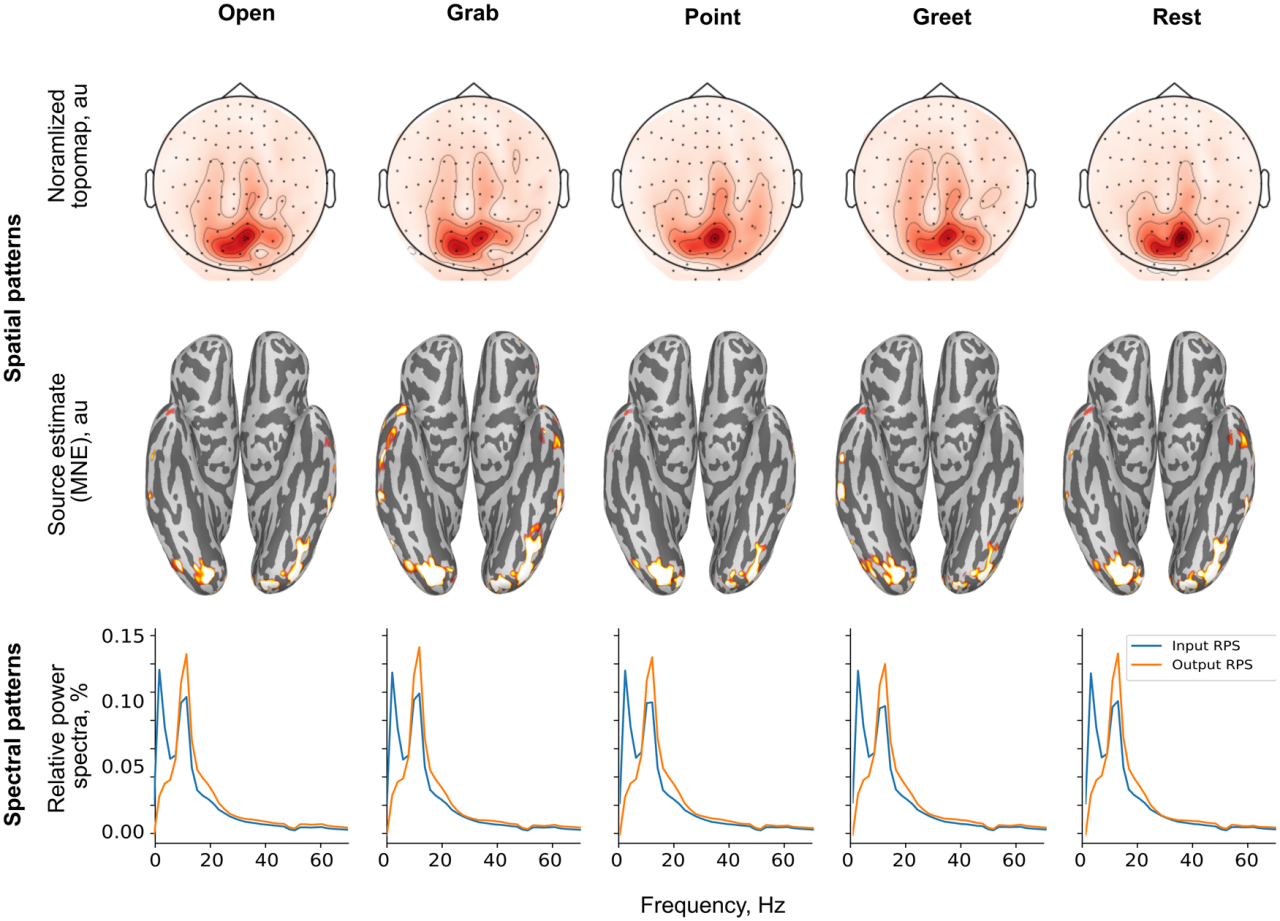
Spatial patterns discriminating the four imagined movements in a single subject (s03).**Top row:**Spatial activation patterns corresponding to the class-conditional mean of each condition in the sensor space (norm of planar gradiometer pairs).**Middle row:**Minimum-Norm source estimates of the spatial activation patterns with the top 10% of the estimate shown.**Bottom row:**Relative power spectra of the MEG signal after the spatial demixing layer (blue), and the estimated spectral fingerprint of the corresponding latent source (orange), both given as a percentage of the total.

## 4 Discussion

The goal of this study was to demonstrate that multiple same-hand movements can be discriminated reliably from the MEG measurements with convolutional neural networks. We used interpretable CNN designed for MEG signals to demonstrate that it may be more suitable for extracting informative features from the MEG signal compared with the popular SVM-based approach. We demonstrate that four real and imagined movements of the same hand can be decoded from unaveraged MEG signals with high accuracy. The obtained accuracies (>70%for real movements and>60%for imagined movements) would be sufficient for inducing and maintaining an operant reinforcement effect, for example, in a rehabilitation setting. Our pattern interpretation analysis verified that the model performance was primarily driven by the known neurophysiological signatures associated with movement preparation and execution, originating primarily from motor and visuomotor areas of the cortex.

### 4.1 Comparison with previous work

An earlier MEG study has demonstrated reliable discrimination of brain responses to button presses performed with the thumb, index, middle, or little finger, yielding an accuracy of 57% for this four-class decoding problem. In the same study, the classification accuracy using EEG was 43% (Quandt et al., 2012). Importantly, the authors demonstrated that the pre-movement brain activity contained information about the specific finger to be moved.

Sugata et al. (2012b)were able to decode three movements (grasp, pinch, and elbow flexion) from MEG signals with 66% accuracy. Importantly, the authors identified three distinct time windows around the movement onset containing patterns of brain activity relevant to the discrimination between the three movements. In our study, we focused on movements performed using the hand only and instructed our subjects to keep the elbow fixed in order to minimize potential contamination of the MEG signal by artifacts resulting from subtle body movements.

Ofner et al. (2017)reported a similar level of accuracy for five sustained same-hand movements (hand open, palmar grasp, lateral grasp, pronation, and supination) when decoding EEG from healthy volunteers (55% for the real movements, and 27% for the imagined movements) as well as from patients with a cervical spinal cord injury (45% for attempted movements).

A recent study byBu et al. (2023)demonstrated that deep convolutional neural networks can achieve remarkably high performance when discriminating executed movements in a three-class rock-paper-scissors task.

In our study, we performed a five-class classification of four naturalistic movements (real and imagined) and the rest condition and reached a higher accuracy than in the previous studies. This increase in accuracy was achieved by combining (1) an experimental paradigm focused on realistic, goal-directed movements, and (2) a convolutional neural network-based decoder developed specifically for MEG signals. Optimizing the experimental design to match the properties of the decoding algorithm allowed us to extract the brain signatures associated with movement preparation, execution (or imagery), and observation and to combine them efficiently for robust classification performance.

Inspecting the confusion matrices (Fig. 3) revealed that the*rest*class proved easier to discriminate from all the other movement classes. Moreover, misclassification between different movements was more likely to occur between pairs of movements which both involved either extension (*open – greet*) or flexion (*grab – point*) of the hand, possibly due to a greater overlap between their respective cortical representations.

In this study, we also investigated the patterns informing the classification and compared them with the results obtained with conventional MEG analysis. Standard time–frequency analysis of our data confirmed robust modulations of the previously known neural signatures associated with both the real and imagined movements in our experiment. However, this analysis could not reveal any notable differences between the different types of movements. Nevertheless, our decoding approach allowed us to discriminate those movements with high accuracy. Spatial and temporal properties of such discriminative features were in line with those obtained using the standard analysis and corresponded to known signatures of movement preparation, imagery, and execution reported previously (Fukuma et al., 2018;Kennedy et al., 2011;Kobler et al., 2020;Magnuson & McNeil, 2021;Sugata et al., 2012a,^2016^;Turella et al., 2016;Wheaton et al., 2005). The passive viewing experiment that we performed as a control further confirmed that these results were not driven by the brain activity evoked by the visual stimuli alone.

### 4.2 Limitations

This study reports the results of the online decoding analysis of the MEG data and thus, may represent an overly optimistic performance estimation compared with the real-time BCI setting for a number of reasons. First, we used several preprocessing steps that are not directly applicable in a real-time BCI setting. For example, tSSS, manual removal of cardiac and oculomotor components, and hyperparameter search. Although analogous automated procedures are reported in the literature, and LFCNN was shown to work in real-time, applying the full-scale real-time analysis to these data is out of the scope of the present study. Second, we used a time-consuming hyperparameter search in combination with cross-validation, which may result in an overestimation of the actual predictive performance. However, in our experiment model performance on the held-out test set was comparable to the cross-validation accuracy the optimal hyperparameter values were identified using the whole dataset.

While LFCNN had outperformed the popular SVM classifier, it is quite likely that even higher performance can be achieved using more powerful models. For example, applying VARCNN (Zubarev et al., 2019) to the same dataset yielded marginally higher cross-validation and out-of-sample performance (seeSupplementary Table S1).

Similarly, this analysis employed healthy volunteers. Future research should focus on determining to which extent these findings generalize to various patient populations.

In both of our control experiments: decoding based on passiveviewing of the visual cues (1) and the accelerometer signal in the imagery condition (2), we observed that the de-facto chance levels differed significantly from the theoretical chance level of 20% for a five-class problem, and were generally corresponding to the chance level of p = 0.001 of a four-class problem (33%) after taking into the account the limited sample size for each subject (n = 300) (Combrisson & Jerbi, 2015). We thus argue that such control procedures may yield a more adequate and conservative estimate of the chance level because they involve testing the decoding model and the experimental design directly against the null hypothesis of (1) responses to the same visual sequence without performing the task or associated oculomotor activity and (2) signals where the informative activity should not be present according to the experimental design. Although one cannot guarantee that, for example, oculomotor artifacts were completely suppressed by removing the corresponding ICA, direct comparisons of the decoding performance with that in the passive viewing control task allow us to argue that our results were primarily driven by the neural activity. To our knowledge, this is the first time that such control procedures are applied to MEG decoding experiments to control for possible confounding factors. We encourage using similar designs to ensure better replicability of future research. These procedures may not, however, fully exclude all possible contributions from non-neural sources including those from, for example, residual body or head movements even after applying the movement compensation. While one may argue that in a practical setting, the system may benefit from these confounding factors, over-relying on such secondary signals can be detrimental to the goal of inducing neuroplasticity.

Our across-subject decoding experiments indicated that the observed activity patterns allowing efficient discrimination in a within-subject setting fail to generalize when applied to held-out subjects. One explanation of these negative results could be that the cortical representation areas of the same-limb movements could be very compact and thus their spatiotemporal patterns are even more susceptible to inter-individual differences in functional neuroanatomy. This interpretation is supported by the fact that the model was able to perform significantly above the chance level on the pooled validation data from the seen subjects. This indicates that the representational capacity of the classifier was still sufficient to learn efficiently from multiple combined subjects. The fact that it failed to generalize to the held-out subjects, however, suggests that spatiotemporal representations of different movements may vary considerably across individuals. While it is not unlikely that across-subject decoding can yield positive results, we believe that demonstrating that convincingly would require collecting data from a much larger number of participants.

While generally in line with previous findings, our pattern interpretation analysis approach intriguingly demonstrated the considerable contribution of the occipital sources to the discrimination between the imagined movements. We emphasize that patterns that discriminative models use to make their predictions should not be interpreted directly in terms of the underlying neural sources until the validity of these techniques receives substantial experimental support. Thus, here we limit the interpretation of these findings to confirming the non-artifactual nature of the performance of our model and refer the reader toHaufe et al. (2014)for a general description of the problem and toWeichwald et al. (2015)for careful analysis of the causal interpretation of such findings.

### 4.3 Implications

Taken together our results suggest that MEG-based BCIs can be used efficiently in a rehabilitation setting to train multiple hand movements with accuracy, sufficient to induce and maintain operant reinforcement. While this study focuses on healthy volunteers, these results look promising enough to encourage similar studies in patient populations. Machine-learning models used in our study were already shown to be applicable in a real-time setting (Zubarev et al., 2019). In the future, similar studies using on-scalp MEG may result in even more robust BCI systems due to the higher spatial resolution of such systems.

## Supplementary Material

Supplementary Material

## Data Availability

The code used to reproduce the main result using the MNEflow package is publicly available at Aalto University Version Control Repository^1^and is included in the Supplementary Material. According to Finnish legislation, MEG signals are regarded as personal data and cannot be made publicly available even if pseudonymized. They can, however, be shared with named parties upon request. We now state in the manuscript that the data can be made available to researchers upon request to the corresponding author.
