# Supplementary material for: Robust discrimination of multiple naturalistic same-hand movements from MEG signals with convolutional neural networks
Source: Imaging Neurosci (Camb). 2024 May 20;2:imag-2-00178. doi: 10.1162/imag_a_00178 (PMC12247546; doi:10.1162/imag_a_00178)
Supplement: Supplementary Material [file imag_a_00178-supp.pdf]

Supplementary material for: Robust discrimination of multiple naturalistic same-hand movements from MEG signals with convolutional neural networks

Ivan Zubarev[a,*], Mila Nurminen[a], Lauri Parkkonen[a,b]

[a] *Department of Neuroscience and Biomedical Engineering, Aalto University School of Science, FI-00076 Espoo, Finland*
[b] *Aalto NeuroImaging, Aalto University, FI-00076, Espoo, Finland*

Table 1: Categorical accuracy in within-subject decoding with RBF-SVM and VARCNN. The CV accuracies are from nine-fold cross-validation.

	Movement				Imagery			
	SVM		VARCNN		SVM		VARCNN	
Subject	CV	TEST	CV	TEST	CV	TEST	CV	TEST
s01	72.1%	63.3%	71.6%	74.7%	62.6%	63.3%	65.4%	69.3%
s02	60.4%	53.3%	71.6%	67.4%	42.2%	40.0%	50.0%	63.0%
s03	78.5%	73.3%	77.8%	69.3%	64.8%	70.0%	72.0%	57.4%
s04	65.6%	80.0%	71.0%	68.9%	49.6%	60.0%	54.0%	55.2%
s05	60.9%	51.5%	67.9%	64.3%	58.2%	45.5%	65.3%	60.3%
s06	53.9%	33.3%	71.8%	64.3%	41.4%	51.5%	41.8%	49.8%
s07	75.1%	72.7%	86.4%	78.5%	68.4%	63.6%	75.9%	72.4%
s08	68.0%	78.8%	69.9%	74.4%	59.9%	57.6%	63.0%	57.2%
s09	67.0%	75.8%	72.7%	69.7%	52.9%	54.5%	62.6%	55.2%
s10	62.6%	57.6%	76.4%	80.5%	42.8%	39.4%	64.8%	69.0%
MEAN	66.4%	64.0%	73.7%	71.2%	54.3%	54.5%	61.5%	60.9%
STD	7.0%	14.2%	5.1%	5.3%	9.4%	9.9%	9.7%	7.0%

*Corresponding author ivan.zubarev@aalto.fi

Table 2: Tested and optimal hyperparameter values for VARCNN classifier.

Parameter	Tested	Final
number of latent components	32	32
kernel size	$32, 64, 128, 256$	128
pooling factor	$16, 32, 64, 128$	32
pooling stride	$8, 16, 32, 64$	32
pooling type	max, avg	avg
non-linear activation function	$ReLU$	$ReLU$
drop-out rate	$0.25, 0.5$	0.5
l_1-penalty	$3 \cdot 10^{-3}$, $3 \cdot 10^{-4}$, $3 \cdot 10^{-5}$	$3 \cdot 10^{-4}$
minibatch size	$5, 25, 50$	50
training iterations per epoch	$25, 50, 100$	25
learning rate	$1 \cdot 10^{-4}$, $3 \cdot 10^{-4}$	$3 \cdot 10^{-4}$

Table 3: Categorical accuracy in within-subject decoding with LF-CNN between the 4 movement classes. The CV accuracies are from nine-fold cross-validation.

	Movement		Imagery		Passive Viewing	
Subject	CV	TEST	CV	TEST	CV	TEST
s01	66.9%	63.7%	70.4%	61.6%	41.5%	36.5%
s02	70.9%	72.4%	61.3%	54.6%	28.4%	28.4%
s03	74.9%	68.9%	66.6%	61.4%	40.2%	35.9%
s04	65.2%	74.4%	58.0%	59.3%	53.9%	52.7%
s05	60.0%	61.1%	64.8%	49.4%	35.3%	41.0%
s06	66.9%	65.5%	40.2%	41.3%	37.7%	20.9%
s07	86.2%	80.3%	74.5%	70.9%	59.6%	53.2%
s08	72.1%	73.9%	68.0%	60.4%	38.3%	38.9%
s09	68.5%	69.8%	55.5%	67.9%	37.0%	33.3%
s10	70.0%	72.0%	57.3%	59.5%	33.9%	30.7%
MEAN	70.2%	70.2%	61.6%	58.6%	40.6%	37.2%
STD	6.6%	5.4%	9.2%	8.1%	8.9%	9.6%

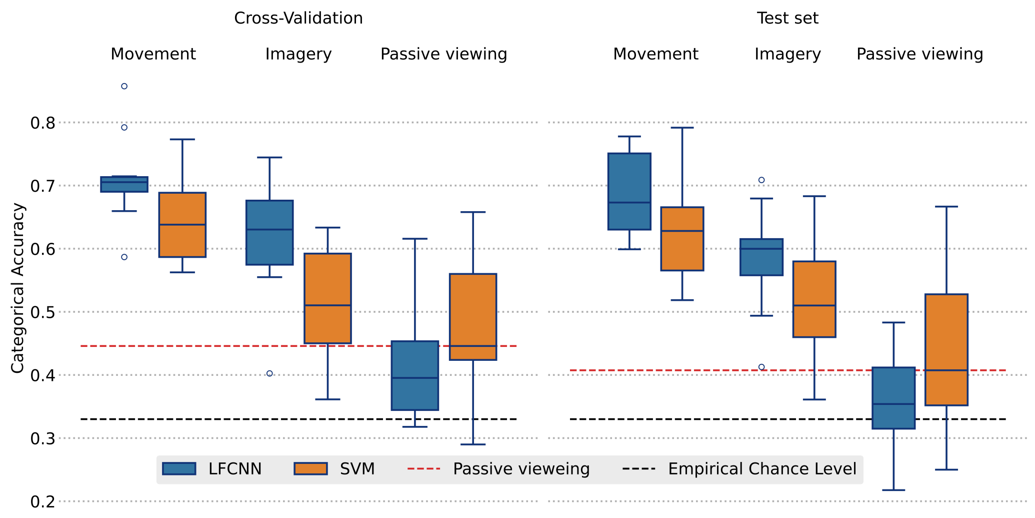

Figure 1: Within-subject (9-fold nested cross-validation) classification results. The box height indicates the interquartile range. Whiskers indicate the full data range, excluding a single outlying data point. Horizontal lines inside the box indicate the median. Empirical chance level (black dashed line) for a 4-class problem with n=300. The de-facto chance level (red dashed line) was defined as the mean cross-validation accuracy obtained in the control passive viewing experiment.

```
import mne
import numpy as np
import tensorflow as tf
import os
import mneflow

datas = []
subs = [''.join(['s', '{:02d}'.format(i)])
        for i in range(1, 11)]

experiment = 'img'
sensetype = 'grad'

path = '~\\data\\wrist\\'

event_ids = {'open':'1', 'grab':'2',
             'point':'4', 'greet':'8',
             'rest':'16',
             'iopen':'101', 'igrab':'102',
             'ipoint':'104', 'igreet':'108'}
```

```


#%%
reimport = True
for sub in subs:
    epochs = []
    if reimport:
        #Import from raw files and preprocess
        if experiment in ['img']:
            conds = ['move', 'imag']
            tmin, tmax = -1., 1.
            epochs_codes = ['101', '102', '104', '108', '16']
            fnames = ["_".join([sub, "wrist", cond, str(j),
                              "tsss_mc_raw.fif"])
                          for j in range(1,4)
                          for cond in conds]

        elif experiment in ['mov']:
            conds = ['move', 'imag']
            tmin, tmax = -1., 1.
            epochs_codes = ['1', '2', '4', '8', '16']
            fnames = ["_".join([sub, "wrist", cond, str(j),
                              "tsss_mc_raw.fif"])
                          for j in range(1,4)
                          for cond in conds]

        elif experiment == 'ctrl':
            conds = ['control']
            tmin, tmax = -0.2, 0.8
            epochs_codes = ['201', '202', '204', '208', '216'
   ]
            fnames = ["_".join([sub, "wrist", cond,
                             "tsss_mc_raw.fif"])
                        for cond in conds]

        else:
            print("Unknown experiment {}".format(experiment))
            break

        for fname in fnames:
            raw = mne.io.RawFIF(path+fname, preload=True)
            events = mne.find_events(raw,
                                     stim_channel='STI101',
                                     output='onset',
                                     min_duration=.003)
```

```
            raw.pick_types(meg=sensetype)
            raw.notch_filter(np.arange(50., 100, 50.),
                             notch_widths=1.)
            raw.filter(l_freq=.1, h_freq=90, method='iir')
            ep = mne.Epochs(raw, events,
                            tmin=tmin, tmax=tmax,
                            decim=2.)
            epochs.append(ep)
            del raw, ep

        epochs = mne.concatenate_epochs(epochs)
        epochs = epochs[epochs_codes]
        ind = list(event_ids.values())

    import_opt = dict(fs=500,
                      path=path,
                      data_id='_'.join([sub,
                                        sensetype,
                                        experiment,
                                        'test']),
                      input_type='trials',
                      overwrite=False,
                      n_folds= 9,
                      test_set = 'holdout',
                      target_type='int',
                      scale=True,
                      scale_interval=None,
                      crop_baseline=False,
                      decimate=None)

    meta = mneflow.produce_tfrecords(epochs, **import_opt)
    dataset = mneflow.Dataset(meta,
                              train_batch=50,
   class_subset=class_subset)
    lf_params = dict(n_ls=32,
                      filter_length=32,
                      pooling = 64,
                      nonlin = tf.nn.relu,
                      stride = 32,
                      padding = 'SAME',
                      dropout = .5,
                      model_path = import_opt['path'],
                      l1_lambda=4e-3,
                      l1_scope=['tconv', 'fc', 'dmx'],
```

```
                        pool_type='avg')

    meta.update(model_specs=lf_params)

    model = mneflow.models.LFCNN(meta)
    model.build(learn_rate=3e-4)

    model.train(n_epochs=500, eval_step=25,
                early_stopping=10, mode='cv',
                collect_patterns=True)

    model.save()
    model.plot_combined_pattern(sensor_layout="Vectorview-
grad")
```

Listing 1: Code using MNEflow software v0.5.6.

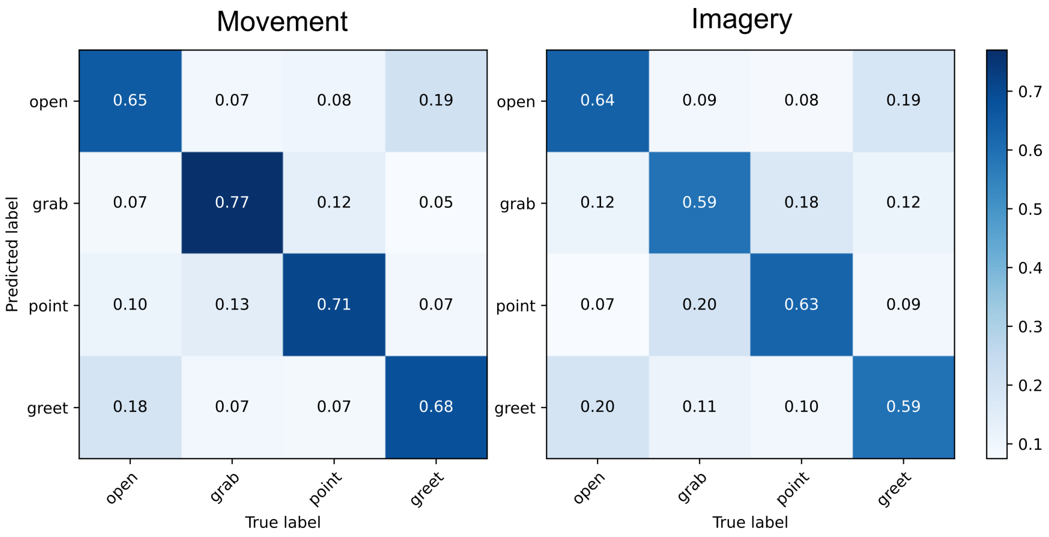

Figure 2: Normalized confusion matrices for cross-validation performance of LFCNN classifier using only four movement classes in movement and imagery experiments (combined from all subjects and all folds)

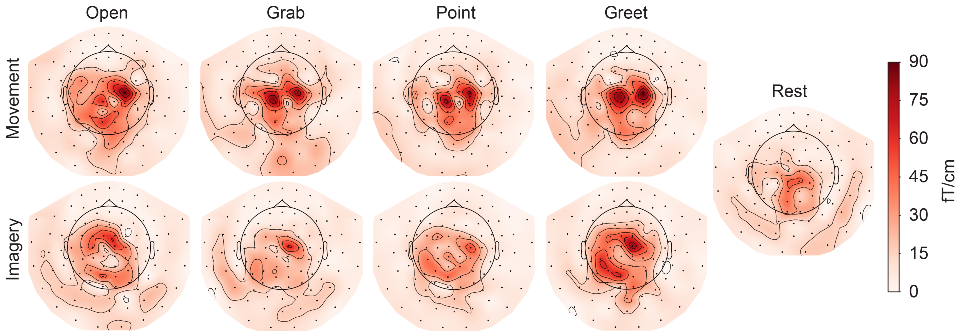

Figure 3: Topographical distribution of task-related evoked fields for s07. Activity is averaged from one second before to one second after the task onset.
